# Natural Compounds for the Treatment of Psoriatic Arthritis: A Proposal Based on Multi-Targeted Osteoclastic Regulation and on a Preclinical Study

**DOI:** 10.2196/resprot.7636

**Published:** 2017-07-11

**Authors:** Shiqiang Deng, Jianwen Cheng, Jinmin Zhao, Felix Yao, Jiake Xu

**Affiliations:** ^1^ Molecular Laboratory School of Biomedical Sciences The University of Western Australia Perth Australia; ^2^ Research Centre for Regenerative Medicine and Guangxi Key Laboratory of Regenerative Medicine Guangxi Medical University Nanning China; ^3^ Department of Orthopaedic Surgery The First Affiliated Hospital of Guangxi Medical University Nanning China

**Keywords:** arthritis, osteoclast, RANKL signaling pathways, anti-psoriatic pharmacotherapy (APP), multi-target, natural compounds, in silico screening, preclinical study

## Abstract

**Background:**

Psoriatic arthritis (PsA) is a chronic inflammatory arthritis affecting approximately 2% to 3% of the population globally, and is characterized by both peripheral articular manifestations and axial skeletal involvement. Conventional therapies for PsA have not been fully satisfactory, though natural products (NPs) have been shown to be highly effective and represent important treatment options for psoriasis. PsA is a multigenic autoimmune disease with both environmental and genetic factors contributing to its pathogenesis. Accordingly, it is likely that the use of natural compounds with a multi-targeted approach will enable us to develop better therapies for PsA and related disorders.

**Objective:**

PsA, either on joint damage or on bone erosion, has been shown to respond to anti-psoriatic pharmacotherapy (APP), APP-like NPs, and their natural compounds. This study aims to uncover specific natural compounds for improved PsA remedies. Specifically, by targeting bone erosion caused by increased osteoclastic bone resorption, we aim to predict the key signaling pathways affected by natural compounds. Further, the study will explore their anti-arthritis effects using an in silico, in vitro, and in vivo approach. Following the signaling pathway prediction, a preclinical efficacy study on animal models will be undertaken. Collectively, this work will discover lead compounds with improved therapeutic effects on PsA.

**Methods:**

We hypothesize that 9 potential APP-like NPs will have therapeutic effects on arthritis via the modulation of osteoclast bone resorption and signaling pathways. For in silico identification, the Latin name of each NP will be identified using the Encyclopedia of Traditional Chinese Medicine (Encyclopedia of TCM). The biological targets of NPs will be predicted or screened using the Herbal Ingredients' Targets (HIT) database. With the designed search terms, DrugBank will be used to further filter the above biological targets. Protein ANnotation THrough Evolutionary Relationship (PANTHER) will be used to predict the pathways of the natural compound sources. Subsequently, an in vitro sample preparation including extraction, fractionation, isolation, purification, and bioassays with high-speed counter-current chromatography-high-performance liquid chromatography-diode array detection (HSCCC-HPLC-DAD) will be carried out for each identified natural source. In vitro investigations into the effect of NPs on osteoclast signaling pathways will be performed. The experimental methods include cell viability assays, osteoclastogenesis and resorption pit assays, quantitative reverse transcription polymerase chain reaction (RT-PCR), western blot, and luciferase reporter gene assays. Finally, an in vivo preclinical efficacy on a collagen-induced arthritis rat model will be carried out using a treatment group (n=10), a control group (n=10), and a non-arthritis group (n=10). Main outcome measure assessments during intervention include daily macroscopic scores and a digital calipers measurement. Post-treatment tissue measurements will be analyzed by serological testing, radiographic imaging, and histopathological assessment.

**Results:**

Studies are currently underway to evaluate the in silico data and the in vitro effects of compounds on osteoclastogenesis and bone resorption. The preclinical study is expected to start a year following completion of the in silico analysis.

**Conclusions:**

The in silico rapid approach is proposed as a more general method for adding value to the results of a systematic review of NPs. More importantly, the proposed study builds on a multi-targeted approach for the identification of natural compounds for future drug discovery. This innovative approach is likely to be more precise, efficient, and compatible to identify the novel natural compounds for effective treatment of PsA.

## Introduction

Psoriatic arthritis (PsA) is a chronic inflammatory arthritis characterized by both peripheral articular manifestations and axial skeletal involvement [1-3]. It affects approximately 2% to 3% of the population globally, with the majority of patients experiencing a chronic, progressive course of disease developing to a destructive, disabling form of arthritis over time [1]. Conventional therapies for psoriasis have not been fully satisfactory and natural products (NPs)—anecdotally known to be highly effective (though with little formal scientific research evidence)—represent important treatment options for psoriasis [4-8]. PsA is considered a systemic inflammatory disease with multifactorial (environmental and genetic) factors contributing to its pathogenesis. Accordingly, a lead compound which simultaneously effects different targets would be ideal for the improved treatment of PsA [4,9]. The practice of combining NPs into formulations, a common method in Chinese medicine, could prove to be an effective alternative approach for the development of multi-compound, multi-target therapies [10].

For PsA, joint damage results from the release of the direct bone-resorbing factors osteoclasts (OCs) and metalloproteinases. An increased frequency of osteoclast precursor (OCP) is found in most patients with PsA and correlates with the extent of radiographic damage observed in affected patients. In osteoclastogenesis, peripheral OCPs and cluster of differentiation (CD) 14^+^ monocytes differentiate to OCs [11]. Disordered osteoclastogenesis leads to altered bone remodeling causing bone erosion and joint damage, represented by increased osteoclast precursors numbers, radiographic damage scores, and disease activity index [3,12]. Typically, this correlates with increased serum levels of the following cellular biomarkers: (1) macrophage-colony stimulating factor (M-CSF); (2) tumor necrosis factor alpha (TNF-α); (3) osteoprotegerin (OPG); (4) receptor activator of nuclear factor-κB ligand (RANKL); (5) CD14^+^/CD11b^+^/CD16^+^; and (7) interleukin (IL-23/IL-17) [3,11,12]. Accordingly, our research direction will focus on the impact of NPs on the above biomarkers, osteoclastogenesis, and joint damage/erosion associated with PsA.

There exists an intrinsic connection between psoriasis and PsA. PsA, either on joint damage or on bone erosion, may respond to anti-psoriatic pharmacotherapy (APP), APP-like NPs, and their natural compounds. A psoriasis study recently developed a novel approach for the identification of promising NPs for psoriasis therapy based on an extensive literature review of current clinical evidence available, followed by an in silico screening of biological targets for APP and drug development. This approach extends beyond psoriasis and can be applied to similar diseases with multifactorial causes for which multi-compound, multi-target therapies are emerging as the therapeutic norm. This approach has been successfully applied in psoriasis and generated robust preliminary data identifying potential NP targets and pathways [13-17]. We have also previously undertaken in vitro and in vivo studies screening for natural compounds that affect osteoclast differentiation and osteoclast-mediated osteolysis, and identified NPs which may have therapeutic potential for the treatment of bone lytic disorders [18-22].

With this project, we aim to (1) identify specific lead natural compounds with therapeutic effects on PsA and/or other autoimmune joint disorders with a multi-target in silico, in vitro, and in vivo approach; (2) provide an overview of the relevant signaling pathways and mechanisms of action; and (3) present therapeutic targets and preclinical efficacy evaluation of APP-like NPs and their natural compounds.

## Methods

### Preliminary Data

Previous work included the development of a novel approach to identify promising NPs for psoriasis treatment based on available clinical evidence followed by in silico screening for biological targets for APP and drug development. Using this combined approach, 9 APP-like NPs have been identified as promising candidates for psoriasis therapy (Table 1) [13-17].

**Table 1:** Promising natural products for psoriasis (N=12) including 9 anti-psoriatic pharmacotherapy-like natural products.

Scientific name	Study design	Administration	APP^a^-like NPs^b^
*Sophora flavescens*	NPM^c^ and APP vs APP	External	Yes
*Cnidium monnieri*	NPM and APP vs APP	External	No
*Dictamnus dasycarpus*	NPM and APP vs APP	External	No
*Borneolum syntheticum*	NPM and APP vs APP	External	No
*Aloe vera*	Single NP vs APP/placebo	External	Yes
*Indigo naturalis*	Single NP vs APP/placebo	External	Yes
*Camptotheca acuminata*	Single NP vs APP/placebo	External	Yes
*Mahonia aquifolium*	Single NP vs APP/placebo	External	Yes
*Sophora flavescens*	NP formula vs APP/placebo	External	Yes
*Lithospermum erythrorhizon*	NP formula vs APP/placebo	External	Yes
*Oldenlandia diffusa*	NP formula vs APP/placebo	Internal	Yes
*Rehmannia glutinosa*	NP formula vs APP/placebo	Internal	Yes
*Salvia miltiorrhiza*	NP formula vs APP/placebo	Internal	Yes

^a^APP: anti-psoriatic pharmacotherapy.

^b^NP: natural product.

^c^NPM: natural product medication.

**Figure 1:**
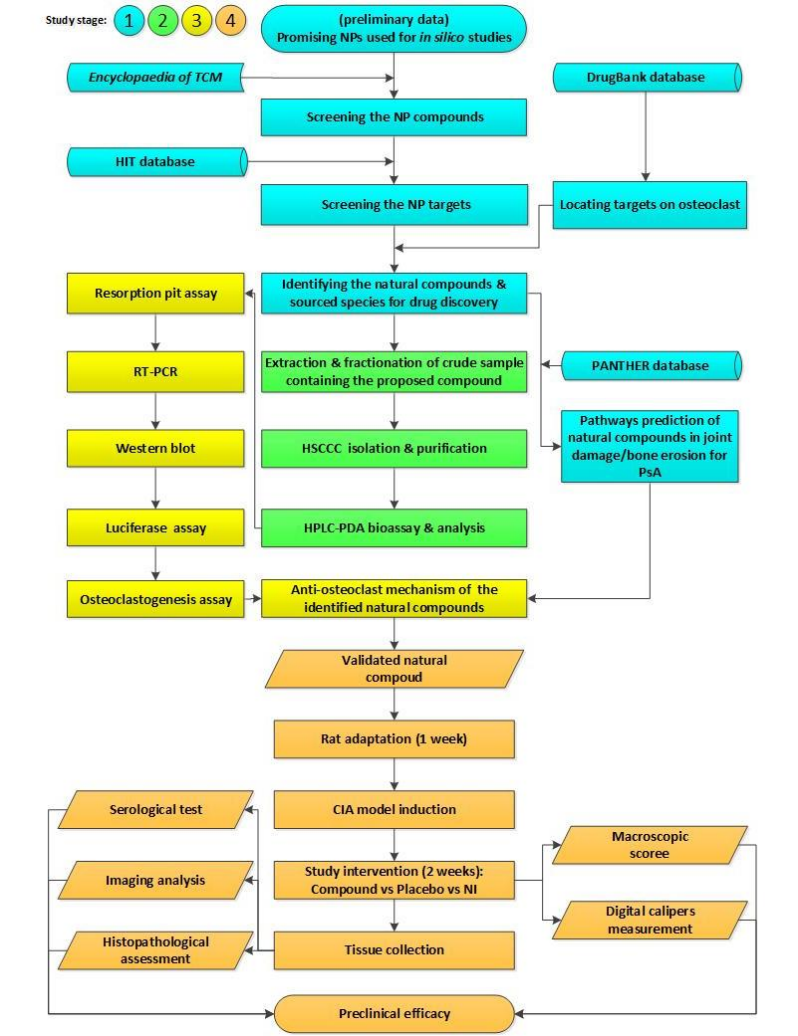
Flowchart of study progression. NI: non-intervention, NP: natural product, PsA: psoriatic arthritis, CIA: collagen-induced arthritis, TCM: traditional Chinese medicine.

The effect of the above NPs on bone erosion and arthritis are still unknown. We hypothesize that these NPs will have therapeutic effects on arthritis via the modulation of osteoclastic bone resorption and signaling pathways. Therefore, the aims of the study consist of the following 4 specific parts: (1) in silico compound identification; (2) in vitro sample preparation; (3) in vitro mechanism investigation; and (4) in vivo preclinical efficacy evaluation (Figure 1).

### In Silico Identification of Prospective Natural Compounds

**Figure 2:**
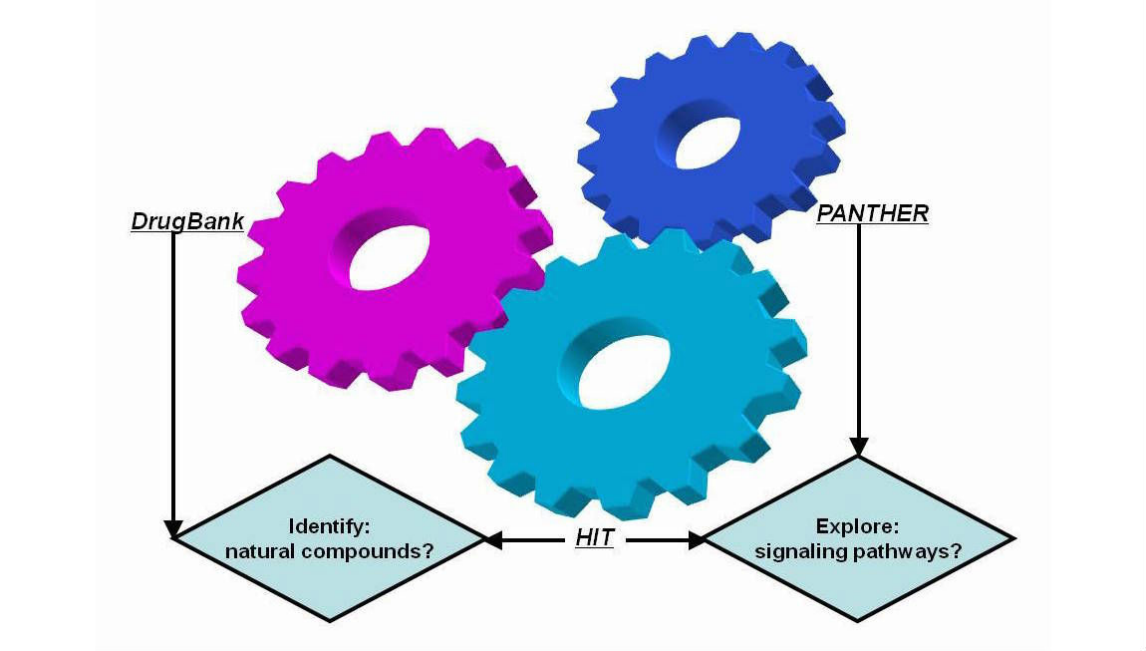
Target-directed in silico identification.

The preliminary data will return a shortlist of promising NPs for an in silico molecular investigation on PsA. In addition to the Encyclopedia of Traditional Chinese Medicine (Encyclopedia of TCM), the Herbal Ingredients' Targets database (HIT, China), DrugBank (Canada), and Protein ANnotation THrough Evolutionary Relationship (PANTHER, USA) will be applied in the in silico identification procedure (Figure 2). All are available in English [13].

The Encyclopedia of TCM can successively locate the relevant Latin names and the contained chemical compounds with the individual and unique plant code of each promising NP species. Subsequently, HIT can access the relevant biological targets with their individual name identification (ID) and types in an Excel spreadsheet. With a dedicated search term(s) (eg, psoriatic arthritis), DrugBank can induce the reference targets (together with the previous cellular biomarkers) to be further filtered by the above biological targets.

For each identified natural compound, their known protein targets will be entered into the keyword search using the “homo sapiens” setting. For each target, the identified Gene ID will be saved a Notepad (txt) file. One Notepad file is created for each species. This file contains all the Gene IDs for all the known therapeutic targets of all the compounds that are known to be active in the species. Each file will be sequentially uploaded into the Gene List Analysis field in PANTHER. This will report the following 5 aspects in Excel for the particular species: (1) molecular function(s), (2) biological processes, (3) cellular component(s), (4) protein class, and (5) pathway(s). The Excel files will be sorted to identify the most commonly identified pathway for each species. For each of the most commonly identified pathways, all the identified proteins, excluding upstream and downstream proteins, will be entered into Excel. Since the nomenclatures used by PANTHER and by HIT differ, cross-referencing will be undertaken regarding the short and long names used for the proteins in both databases.

### In Vitro Sample Preparation Using the Identified Compound Sources for Drug Development

This stage includes the extraction, fractionation, isolation, purification, and the formation of a series of bioassays for the identified compounds. The extraction method is specific to the nature of the source material and target compound. It typically involves a process of drying, grinding, homogenization, or maceration of the plant. For a pure single compound, the crude extract initially needs to be fractionated into various discrete fractions containing compounds in similar polarities or molecular sizes. NP isolation is also subject to the nature of the target compound(s) presented in the crude extract or fraction. Part sample compounds may be purchased from the market or supported by the relevant group. Qualitative chemical tests, preliminary thin-layer chromatography (TLC), and/or high performance liquid chromatography-photodiode array hyphenated technique (HPLC-PDA) can be used to obtain spectral profiles from molecular mixtures or chromatographically separated samples [23]. For isolation and purification, an efficient on-line purity monitoring strategy based on on-line coupling of high-speed counter-current chromatography (HSCCC) with high performance liquid chromatography-diode array detection (HPLC-DAD) will be performed. In summary, this part of project consists of the preparation of a crude extract, preparation of a 2-phase solvent system, HSCCC separation, and HPLC-DAD purity analysis of counter-current chromatography (CCC) peak fractions [24].

### In Vitro Investigation of Drug Candidates

**Figure 3:**
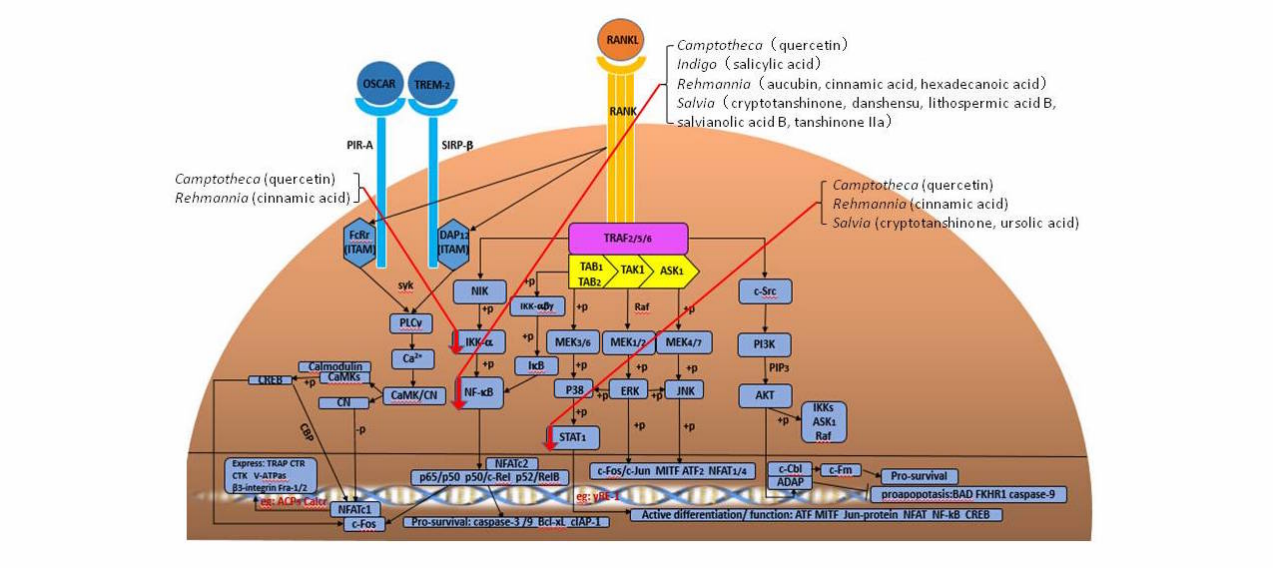
Natural compound inhibitors of the RANKL pathway.

We will perform in vitro studies on compounds prepared in stage 2 to investigate their effects on osteoclastogenesis and bone resorption (Figure 3). For osteoclast formation, 1×10^4^ bone marrow macrophage (BMM) cells per well will be isolated from the femur and tibiae of 6-week-old C57BL/6 mice and will be cultured in media containing macrophage-colony stimulating factor (M-CSF) (30 ng/mL) and RANKL (100 ng/mL). After a cell viability evaluation, further investigations will be carried out using resorption pit assays, quantitative reverse transcription polymerase chain reactions (RT-PCR), western blot analysis, luciferase reporter gene activity assays, and osteoclastogenesis assays. For statistical analysis, the student Newmane-Keuls test will be applied with mean (SD) for data expression and *P* less than .05 for statistical significance [25].

### Preclinical Efficacy Evaluation Using Animal Models

The collagen-induced arthritis (CIA) model is regarded as the best-studied animal model for PsA [26] and will be used in this study to determine the anti-arthritic effects of NPs (as utilized in previously published work by this laboratory) [19,21].

A total of 30, 9-week old female Dark Agouti (DA) rats will be used with 10 assigned to the non-arthritic control group (C). The remaining 20 rate will be induced following the CIA protocol and will be randomly assigned to either the treatment group (A) or the placebo group (B) when PsA symptoms first appear. Groups A and B will be subject to a subcutaneous injection of the purified natural compound (1 mg/kg in 0.9% saline) or 0.9% saline control every second day from onset of symptoms (clinical score 2 or greater), until tissue collection at day 14. Natural compounds differ in their bioavailability and solubility in water. As such, the proposed dosage and/or administration of may be further adjusted if required. All rats will be raised with water, 0.9% sodium chloride (NaCl) and standard rodent food ad libitum in a 22℃ and 12h illuminated daily environment. Main outcome measures include a daily macroscopic scoring system and digital calipers to measure dorsal to plantar thickness (3 times per week) of each paw. Groups will be compared with (1) serological tests including serum albumin, alanine transaminase (ALT), aspartate transaminase (AST), and bilirubin using a clinical biochemical analyzer; (2) micro-computed tomography (micro-CT) image analysis of hind paws and femora and contact radiographs for both hind feet; and (3) histopathological assessment using tartrate-resistant acid phosphatase (TRAP) staining [27] of serial 5 μm sagittal sections through the digits. The latter requires at least 2 digits randomly taken from each rat. Each digit will have distal interphalangeal (DIP), proximal interphalangeal (PIP), and metatarsophalangeal (MTP) joints intact for scoring by 2 independent observers. Analysis of variance (ANOVA) and Fishers' protected least significant difference (PLSD) tests will be applied for statistical analyses (using StatView 4.0) with *P* values less than .05 indicating statistical significance.

## Results

**Figure 4:**
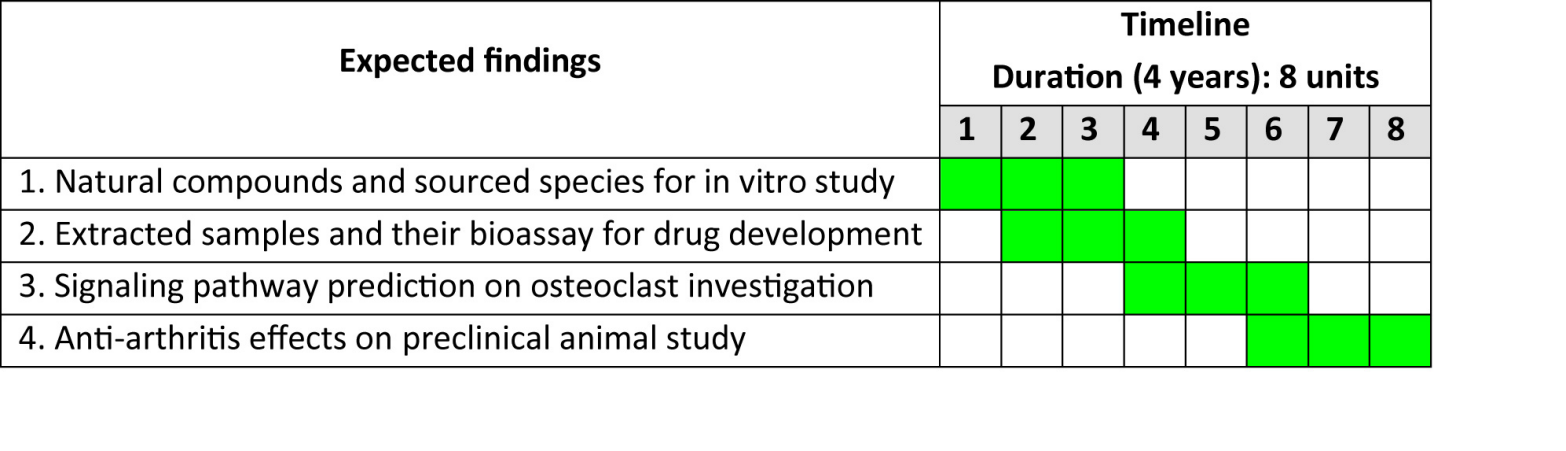
Study timelines. Green squares represent scheduled parts.

This 4-stage study will take approximately 4 years to complete and proposes to discover the effects of natural compounds against PsA through the identification of relevant signaling pathways and preclinical efficacy evaluations using animal models (Figure 4). Currently, we are in the early stages of evaluating the in silico data and detailing the in vitro effects of compounds on osteoclastogenesis and bone resorption. The findings from this research are expected to be published when the proposed studies are fully completed.

## Discussion

### Significance of Findings

Anecdotal evidence suggests that promising NPs are able to act on specific targets regulating osteoclast function and here they constitute the compounds identified for PsA drug discovery. For each identified compound, its extracted sample will be validated in a preparation bioassay such as HPLC. This can directly prove or discount a previous PANTHER prediction by demonstrating the signaling pathways associated with joint damage and/or bone erosion. Further preclinical studies will demonstrate the anti-arthritic effects of validated natural compounds. The principal methods and findings of this project can be further applied in the search of other natural compounds effective against other arthropathies.

### In Silico, Multi-Targeted Approach

The in silico, multi-targeted approach is a target-directed large-scale analysis using multiple databases [13]. It is able to locate biological targets for NPs and APPs. Consequently, the consolidated list of APP targets could be used to screen NPs target lists. This approach could also be used to further identify APP-like NPs by filtering out those targets irrelevant to any of the APPs. This can “fish” out a specific target across NPs and APPs. The identification of protein targets related to a specific natural compound can also reflect its therapeutic molecular mechanisms and hint at potential side effects. However, the number of estimated potential targets on the human genome is huge [28]. Therefore, an in silico model is necessary for large-scale data analysis to elucidate potential protein targets associated with natural compounds (including their formulae) effective against a certain disease.

That the HIT database contains a list of NPs along with their active ingredient makes this model innovative and broadens the scope of searches, ensuring the inclusion of all potentially relevant proteins [13]. An additional novelty is the “APP-like” NP identification and the NP/APP property exploration. With such a database cluster, the NP target list can be matched against potential APP drug targets to identify APP-like NPs. This may provide a direct biological and/or pharmaceutical comparison between NPs and APPs, in terms of their possible effects on specific proteins, for the same disorder.

### The Reliability of In Silico Screening Data

The in silico solution extensively uses frequency effects in target identification, which provides an objective approach to short listing targets and facilitates the reliability of the in silico data to a considerable extent. On the other hand, popular targets often reflect the hot topics previously undertaken in natural product research. Due to a number of studies and reports on natural products in chronic inflammation with PsA, some inflammatory targets may be presented in high frequency within the identified data sets. It is also noted that multiple terms of a single potential target often exist in the above main databases (HIT, DrugBank, and PANTHER) [13]. The variable nomenclature in different databases may impact the reliability of data when locating a crossing target. Structure-based drug docking has emerged as a valuable tool to identify lead compounds and to facilitate drug development.

Following the in silico work, we will undertake in vitro studies and an in vivo CIA preclinical trial to individually validate the anti-arthritic effects of the identified natural compounds which have shown promise to be effective against PsA *.* During this part of the project, we will adopt standardized quality control over procedures, a high performance analysis approach, and employ established animal models to ensure the study’s success and feasibility.

### Conclusion

The in silico rapid approach is proposed as a more general method for adding value to the results of a systematic review of NPs. More importantly, the proposed study builds on a multi-targeted approach for the identification of natural compounds for future drug discovery. This innovative approach is likely to be more precise, efficient, and compatible to identify the novel natural compounds for effective treatment of PsA.

## Abbreviations

APP: anti-psoriatic pharmacotherapy
CD: cluster of differentiation
CIA: collagen-induced arthritis
DAD: diode array detection
Encyclopedia of TCM: Encyclopedia of Traditional Chinese Medicine
HIT: Herbal Ingredients' Targets database
HPLC: high-performance liquid chromatography
HSCCC: high-speed counter-current chromatography
ID: identification
IL: interleukin
NPM: natural product medication
RANKL: receptor activator of nuclear factor-κB ligand
TRAP: tartrate-resistant acid phosphatase

## References

[1] Haroon M, FitzGerald O (2016). Psoriatic arthritis: complexities, comorbidities and implications for the clinic. Expert Rev Clin Immunol.

[2] Myers WA, Gottlieb AB, Mease P (2006). Psoriasis and psoriatic arthritis: clinical features and disease mechanisms. Clin Dermatol.

[3] Xue Y, Jiang L, Cheng Q, Chen H, Yu Y, Lin Y, Yang X, Kong N, Zhu X, Xu X, Wan W, Zou H (2012). Adipokines in psoriatic arthritis patients: the correlations with osteoclast precursors and bone erosions. PLoS One.

[4] Chen X, Decker M (2013). Multi-target compounds acting in the central nervous system designed from natural products. Curr Med Chem.

[5] Kwak HB, Lee BK, Oh J, Yeon J, Choi S, Cho HJ, Lee MS, Kim J, Bae J, Kim SH, Kim HS (2010). Inhibition of osteoclast differentiation and bone resorption by rotenone, through down-regulation of RANKL-induced c-Fos and NFATc1 expression. Bone.

[6] Newman DJ, Cragg GM, Snader KM (2003). Natural products as sources of new drugs over the period 1981-2002. J Nat Prod.

[7] Wang YN, Zhang Y, Wang Y, Zhu DX, Xu LQ, Fang H, Wu W (2014). The beneficial effect of total glucosides of paeony on psoriatic arthritis links to circulating Tregs and Th1 cell function. Phytother Res.

[8] Zhai ZJ, Li HW, Liu GW, Qu XH, Tian B, Yan W, Lin Z, Tang TT, Qin A, Dai KR (2014). Andrographolide suppresses RANKL-induced osteoclastogenesis in vitro and prevents inflammatory bone loss in vivo. Br J Pharmacol.

[9] Chen HS, Qi SH, Shen JG (2017). One-compound-multi-target: combination prospect of natural compounds with thrombolytic therapy in acute ischemic stroke. Curr Neuropharmacol.

[10] Sucher NJ (2013). The application of Chinese medicine to novel drug discovery. Expert Opin Drug Discov.

[11] Paek SY, Han L, Weiland M, Lu C, McKinnon K, Zhou L, Lim HW, Elder JT, Mi Q (2015). Emerging biomarkers in psoriatic arthritis. IUBMB Life.

[12] Dalbeth N, Pool B, Smith T, Callon KE, Lobo M, Taylor WJ, Jones PB, Cornish J, McQueen FM (2010). Circulating mediators of bone remodeling in psoriatic arthritis: implications for disordered osteoclastogenesis and bone erosion. Arthritis Res Ther.

[13] Deng S (2014). Chinese herbal medicine for psoriasis: evaluation of clinical evidence and investigation of the anti-psoriatic effects of specific Chinese medicinal herbs.

[14] Deng S, May BH, Zhang AL, Lu C, Xue CC (2013). Topical herbal medicine combined with pharmacotherapy for psoriasis: a systematic review and meta-analysis. Arch Dermatol Res.

[15] Deng S, May BH, Zhang AL, Lu C, Xue CC (2013). Plant extracts for the topical management of psoriasis: a systematic review and meta-analysis. Br J Dermatol.

[16] Deng S, May BH, Zhang AL, Lu C, Xue CC (2014). Phytotherapy in the management of psoriasis: a review of the efficacy and safety of oral interventions and the pharmacological actions of the main plants. Arch Dermatol Res.

[17] Deng S, May BH, Zhang AL, Lu C, Xue CC (2014). Topical herbal formulae in the management of psoriasis: systematic review with meta-analysis of clinical studies and investigation of the pharmacological actions of the main herbs. Phytother Res.

[18] Huang J, Zhou L, Wu H, Pavlos N, Chim SM, Liu Q, Zhao J, Xue W, Tan RX, Ye J, Xu J, Ang ES, Feng H, Tickner J, Xu J, Ding Y (2015). Triptolide inhibits osteoclast formation, bone resorption, RANKL-mediated NF-қB activation and titanium particle-induced osteolysis in a mouse model. Mol Cell Endocrinol.

[19] Liu Q, Zhao J, Tan R, Zhou H, Lin Z, Zheng M, Romas E, Xu J, Sims NA (2015). Parthenolide inhibits pro-inflammatory cytokine production and exhibits protective effects on progression of collagen-induced arthritis in a rat model. Scand J Rheumatol.

[20] Song F, Zhou L, Zhao J, Liu Q, Yang M, Tan R, Xu J, Zhang G, Quinn JM, Tickner J, Huang Y, Xu J (2016). Eriodictyol inhibits RANKL-induced osteoclast formation and function via inhibition of NFATc1 activity. J Cell Physiol.

[21] Zhou C, You Y, Shen W, Zhu Y, Peng J, Feng H, Wang Y, Li D, Shao W, Li C, Li W, Xu J, Shen X (2016). Deficiency of sorting nexin 10 prevents bone erosion in collagen-induced mouse arthritis through promoting NFATc1 degradation. Ann Rheum Dis.

[22] Zhou L, Liu Q, Yang M, Wang T, Yao J, Cheng J, Yuan J, Lin X, Zhao J, Tickner J, Xu J (2016). Dihydroartemisinin, an anti-malaria drug, suppresses estrogen deficiency-induced osteoporosis, osteoclast formation, and RANKL-induced signaling pathways. J Bone Miner Res.

[23] Sarker SD, Nahar L (2012). An introduction to natural products isolation. Methods Mol Biol.

[24] Zhou T, Zhu Z, Wang C, Fan G, Peng J, Chai Y, Wu Y (2007). On-line purity monitoring in high-speed counter-current chromatography: application of HSCCC-HPLC-DAD for the preparation of 5-HMF, neomangiferin and mangiferin from Anemarrhena asphodeloides Bunge. J Pharm Biomed Anal.

[25] Nie S, Xu J, Zhang C, Xu C, Liu M, Yu D (2016). Salicortin inhibits osteoclast differentiation and bone resorption by down-regulating JNK and NF-κB/NFATc1 signaling pathways. Biochem Biophys Res Commun.

[26] Weitz JE, Ritchlin CT (2013). Mechanistic insights from animal models of psoriasis and psoriatic arthritis. Curr Rheumatol Rep.

[27] Minkin C (1982). Bone acid phosphatase: tartrate-resistant acid phosphatase as a marker of osteoclast function. Calcif Tissue Int.

[28] Zheng CJ, Han LY, Yap CW, Ji ZL, Cao ZW, Chen YZ (2006). Therapeutic targets: progress of their exploration and investigation of their characteristics. Pharmacol Rev.

